# Downregulation of Ezrin Suppresses Migration Potential in Cervical Cancer Cells

**DOI:** 10.3390/ph18010003

**Published:** 2024-12-24

**Authors:** Marta Hałas-Wiśniewska, Wioletta Arendt, Alina Grzanka, Magdalena Izdebska

**Affiliations:** Department of Histology and Embryology, Faculty of Medicine, Collegium Medicum in Bydgoszcz, Nicolaus Copernicus University in Toruń, Karłowicza 24, 85-092 Bydgoszcz, Poland; warendt@cm.umk.pl (W.A.); agrzanka@cm.umk.pl (A.G.); mizdebska@cm.umk.pl (M.I.)

**Keywords:** ezrin, cervical cancer, actin, migration, metastasis

## Abstract

Background: The literature reports that ezrin (EZR) is important as a linker between microfilaments and cellular environments. Moreover, it affects cancer cell migration, but the exact mechanism is not fully understood. In this study, we aimed to investigate the role of EZR in the migration of two different types of cervical cancer cells—from primary lesion (SiHa) and lymph node metastases (HT-3). In addition, we showed for the first time that a reduced EZR protein level affects the cellular response to the routinely used treatment with cisplatin. Methods: The most important stage of the study consisted of conducting a series of tests enabling the assessment of the migration potential of cervical cancer cells without altered EZR expression and with silenced protein expression. Results: Reducing the EZR level resulted in a decrease in the invasive and migration potential of SiHa and HT-3 cells’ inhibition of colony formation, a decrease in adhesive properties, and a strong reorganization of F-actin with a dominance of cells with a mitotic catastrophe phenotype. A lower level of protein significantly reduces the motor skills of SiHa and HT-3 cervical cancer cells. Conclusions: This significantly affects the assessment of EZR as a potential factor that can limit the development of metastases in targeted cancer therapy of cervical cancer.

## 1. Introduction

The ability of primary cancer cells to invade neighboring tissues and then form local or distant metastases is one of the factors determining the high mortality rate of oncological patients. Single cancer cells, changing the phenotype from epithelial to mesenchymal, cross the border of the basement membrane and, thanks to the reorganization of, among others, structures of the actin cytoskeleton, acquire motor skills. This process is called epithelial–mesenchymal transition (EMT) and is associated with a loss of epithelial cell polarity and adhesion. Subsequently, cancer cells migrate and metastasize, and when they reach the new environment, they undergo the reverse process of mesenchymal–epithelial transition (MET) as the initiation of metastatic growth [1,2]. One of the elements of the entire metastatic machine is ezrin (EZR). This protein is the connector between the membrane and the actin cytoskeleton. In addition, it plays a key role in polarization and the course of cell migration and division. It takes part in the formation of protrusions necessary for the cell to obtain motor properties [3,4,5]. Moreover, it plays a significant role in the migration and metastasis of cancer cells by reorganizing the actin cytoskeleton and influencing cell signaling pathways [6]. Numerous scientific studies indicate that EZR is often dysregulated in human cancers. More frequent metastasis and higher mortality of cancer patients are often associated with overexpression of the protein and change of its localization, as well as its abnormal activation. Upregulation of EZR has been identified in various types of cancers, such as breast, colon, and pancreatic cancer [6,7,8]. Therefore, studies presenting EZR as a valuable prognostic marker are justified.

Cervical cancer is classified as the fourth most deadly female oncological disease. For the primary focus, the five-year survival rate is estimated at 92%, while, in the case of regional and distant metastases, this parameter decreases to 59% and 17%, respectively. In addition, 50% of cases are advanced stages of the disease, with new secondary foci in the lymph nodes. These statistics may be related to the invasiveness of this type of cancer, as well as resistance to routinely used methods of treatment, including cytostatics [9].

Therefore, the aim of the project is to determine the importance of EZR in determining the aggressiveness and metastatic potential of cervical cancer cells. Additionally, for the first time, we assess the impact of EZR reduction on the response of cells to cytostatic drugs commonly used to treat this type of cancer.

## 2. Results

### 2.1. Examination of EZR Knockdown in SiHa and HT-3 Cell Lines

**Figure 1:**
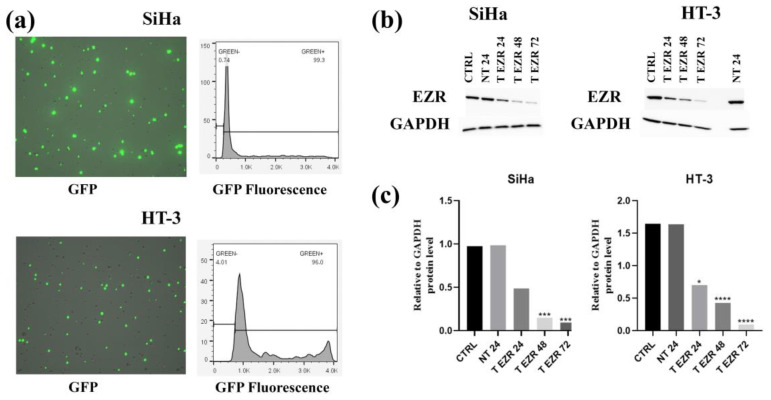
Transfection efficiency of EZR downregulation. (**a**) Representative images of green fluorescence (GFP)-positive cells. Transfection efficiency was estimated based on the percentage of GFP-positive cells in the SiHa and HT-3 cell lines. (**b**) Representative results of Western blots. (**c**) Semi-quantitative densiometric analysis of EZR and GAPDH protein levels in the SiHa and HT-3 cell lines at 24, 48, and 72 h post-transfection. CTRL (cells with control siRNA), NT 24 (cells without transfection after 24 h), T EZR 24 (cells with siRNA against EZR, 24 h after electroporation), T EZR 48 (cells with siRNA against EZR 48 h after electroporation), T EZR 72 (cells with siRNA against EZR 72 h after electroporation), and NT 24 (cells without transfection after 24 h cultured). Statistically significant differences between the NT 24 group and the other groups are marked as ‘*’ *p* < 0.05, ‘***’ *p* < 0.001, and ‘****’ *p* < 0.0001.

Evaluation of the level of GFP fluorescence using a Tali imaging cytometer enabled the assessment of transfection efficiency. The percentage of GFP-positive cells 24 h after electroporation was 99.3% for SiHa cells and 96% for HT-3 cells (Figure 1a). The EZR level was assessed in non-transfected cells (NT), with control plasmid (CTRL) and siRNA against EZR (T EZR) after 24, 48, and 72 h using the Western blot method. We noted no statistical differences in EZR protein levels in non-transfected and CTRL cells for both lines (Figure 1b). In turn, comparative analysis of densiometric results for the SiHa NT and SiHa T EZR cell populations showed statistically significant protein expression knockdown (2.02-fold for 24 h, 6.06-fold for 48 h, and 11-fold for 72 h). We noted a similar result for the HT-3 T EZR cells; when compared to HT-3 NT cells, the level of protein reduction was 2.34-fold, 3.81-fold, and 18-fold after 24, 48, and 72 h, respectively (Figure 1c). Based on the results obtained, 72 h was considered the most effective transfection time and was used in subsequent experiments. Moreover, visualization of EZR by fluorescence in both cervical cancer cell lines also confirmed a weaker signal of this protein in cells (72 h after electroporation) compared to non-transfected cells and with the control plasmid.

### 2.2. The Effect of EZR Downregulation on Basic Cellular Processes in Cervical Cancer Cells

**Figure 2:**
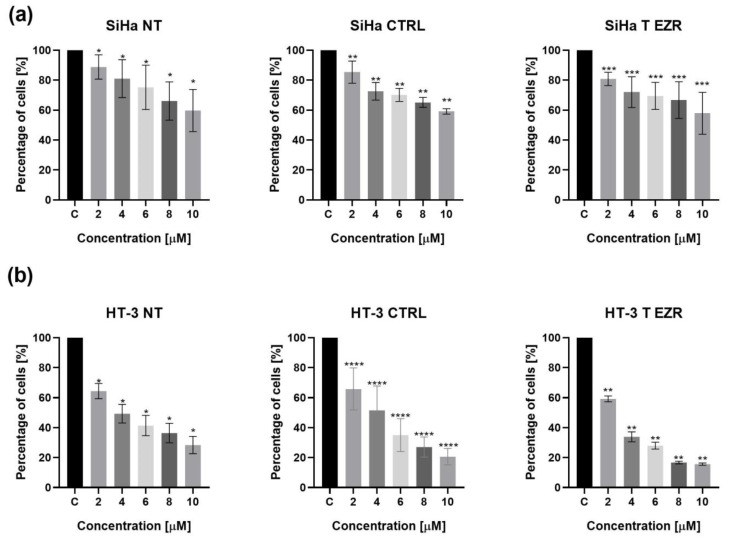
Evaluation of CP cytotoxicity. (**a**) Results of MTT test for the SiHa cell line. (**b**) Results of MTT test for the HT-3 cell line. NT—cells without transfection, CTRL—cells with control siRNA, T EZR—cells with siRNA directed against EZR, and C—untreated cells. 2, 4, 6, 8, and 10—CP doses in µM. Statistically significant differences in compared to C were marked as ‘*’ *p*< 0.05, ‘**’ *p* < 0.01, ‘***’ *p*< 0.001, and ‘****’ *p* < 0.0001.

To investigate whether reducing the level expression of EZR will affect the response of SiHa and HT-3 cells to cytostatics routinely used in therapy, a series of tests was performed to check the basic vital parameters of the cells. The MTT test was the first to be used to estimate the cytotoxicity of CP on NT, CTRL, and T EZR cells of both lines of cervical cancer. Based on our obtained data, we selected dose 8 µM CP for further study. Our selection was based on the results of the primary lesion, which is more resistant to cytostatic drugs than the metastasis. In the case of the assessment of SiHa cell survival, the analysis showed that the EZR T cell population had a similar pattern of CP resistance as NT and CTRL cells. Statistical significance was obtained only between untreated cells and individual doses in all groups. The reduction in the percentage of viable cells for the 8 µM dose was 34% for SiHa NT, 35% for SiHa CTRL, and 34% for SiHa T EZR (Figure 2a). As shown in Figure 2b, 24-h incubation of HT-3 cells, untransfected and transfected with the control plasmid and EZR siRNA, showed a dose-dependent response of the cells to CP. Statistical significance was noted between untreated cells and individual drug doses. Analysis of the results indicated that HT-3 cells with a lower level of EZR expression were more sensitive to drugs. The differences noted between HT-3 NT and HT-3 CTRL were also not statistically significant, contrary to the comparative analysis between HT-3 NT and HT-3 T EZR (Figure 2b).

**Figure 3:**
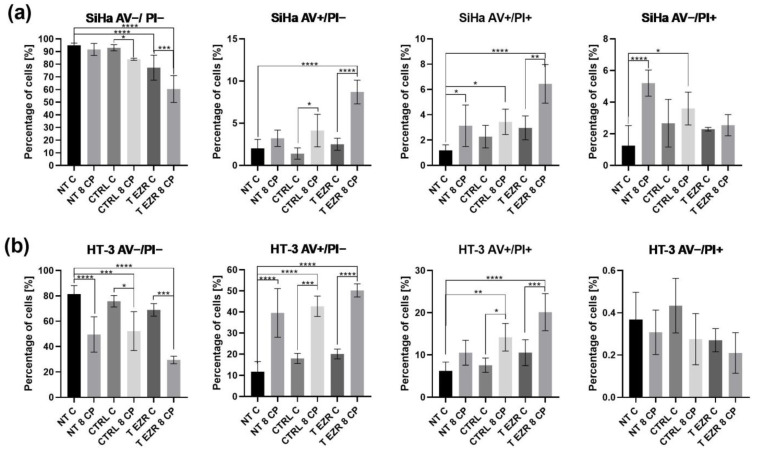
Cell death analysis. (**a**) Results of the AV/PI test for the SiHa cell line. (**b**) Results of the AV/PI test for HT-3 cell line. NT—cells without transfection, CTRL—cells with control siRNA, T EZR—cells with siRNA directed against EZR, C—untreated cells, 8 CP—cells treated with 8 µM CP. Live cells—AV−/PI−, early apoptotic cells—AV+/PI−, late apoptotic cells—AV+/PI+, and necrotic cells—AV−/PI+. Statistically significant differences were marked as ‘*’ *p*< 0.05, ‘**’ *p* < 0.01, ‘***’ *p*< 0.001, and ‘****’ *p* < 0.0001.

The next step was to evaluate the cytostatic treatment of cervical cancer cells without and with reduced EZR levels in the context of cell death induction. The AV/PI double-staining test showed that SiHa NT and SiHa CTRL cells have a similar response to the 8 µM concentration of CP. The highest percentage of early and late apoptotic cells was observed for cells with the knockdown protein level subjected to 24 h incubation with CP (8.7% and 6.44%, respectively) (Figure 3a). Analysis of cell death for the HT-3 cell line showed that cells derived from lymph node metastasis are more sensitive to the drug. The use of 8 µM statistically significantly increased the percentage of apoptotic cells, especially in the T EZR population (early apoptosis—50.24% and late apoptosis—20.14%) (Figure 3b).

**Figure 4:**
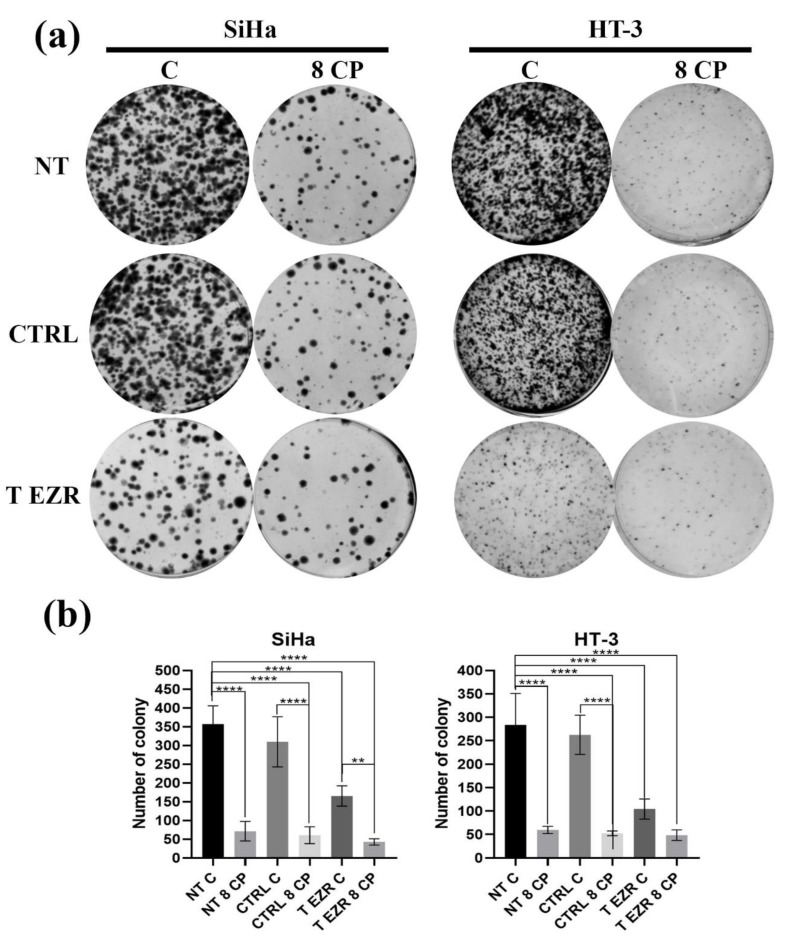
Colony formation assay. (**a**) Representative images of the colony formation assay for SiHa and HT-3 cells. (**b**) Statistical analysis of average results. NT—cells without transfection, CTRL—cells with control siRNA, T EZR—cells with siRNA directed against EZR, C—untreated cells, and 8 CP—cells treated with 8 µM CP. The average results of the tests. Statistically significant differences were marked as ‘**’ *p* < 0.01 and ‘****’ *p* < 0.0001.

As shown in Figure 4a,b, SiHa and HT-3 cells with a knockdown EZR level had a slower colony formation rate compared to non-transfected and with control siRNA SiHa and HT-3 cells. The mean value for untreated SiHa cells was 357 colonies and, for SiHa transfected with the control plasmid, 309 colonies. For the cells with siRNA against EZR, it was in the range of 165 colonies. A decrease in the number of colonies formed was also observed for the second line of cervical cancer cells. In this case, the mean values were 283 colonies for untreated HT-3, 262 colonies for HT-3 transfected with the control plasmid, and 104 colonies for cells with reduced protein levels. Moreover, this effect was enhanced after 24 h of incubation with a cytostatic agent (Figure 4b).

### 2.3. The Effect of EZR Downregulation on the Migration Potential of SiHa and HT-3 Cells

To identify the changes in the migration potential of SiHa and HT-3 cells non-transfected with EZR siRNA and control siRNA, the wound healing test, migration/invasion Transwell assays, and adhesion tests were performed.

**Figure 5:**
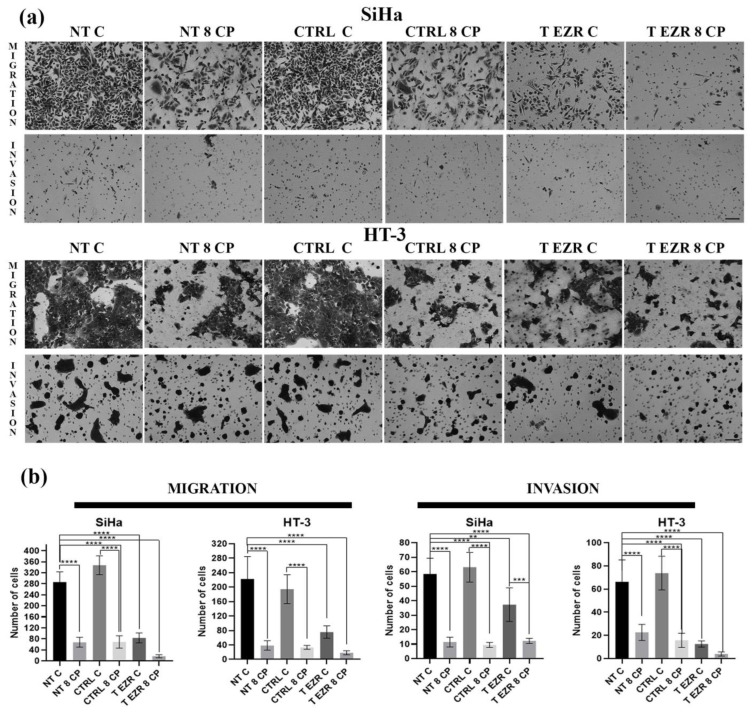
Results of the multipath panel of migration. (**a**) Representative images of the migration Transwell assay and invasion Transwell assay of SiHa and HT-3 cells. Scale bar = 100 µm. (**b**) Statistical analysis of the average results. NT—cells without transfection, CTRL—cells with control siRNA, T EZR—cells with siRNA directed against EZR, C—untreated cells, and 8 CP—cells treated with 8 µM CP. The average results of the tests. Statistically significant differences were marked as ‘**’ *p* < 0.01, ‘***’ *p*< 0.001, and ‘****’ *p* < 0.0001.

**Figure 6:**
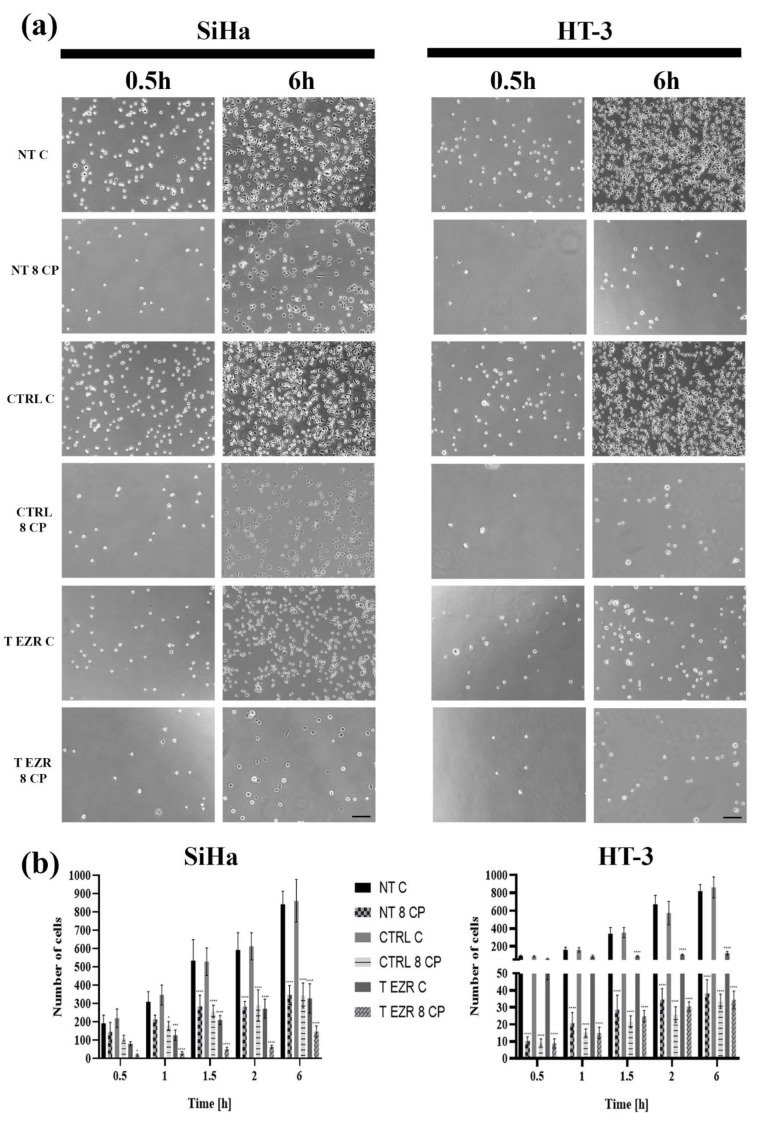
Adhesion assay results. (**a**) Representative images of the adhesion assay after 0.5 h and 6 h of seeding SiHa and HT-3 cells. Scale bar = 50 µm. (**b**) Statistical analysis of the mean results at all time points (0.5, 1, 1.5, 2, and 6 h). NT—cells without transfection, CTRL—cells with control siRNA, T EZR—cells with siRNA directed against EZR, C—untreated cells, and 8 CP—cells treated with 8 µM CP. The average results of the tests. Statistically significant differences in comparison to NT C were marked as ‘*’ *p*< 0.05, ‘***’ *p*< 0.001, and ‘****’ *p* < 0.0001.

**Figure 7:**
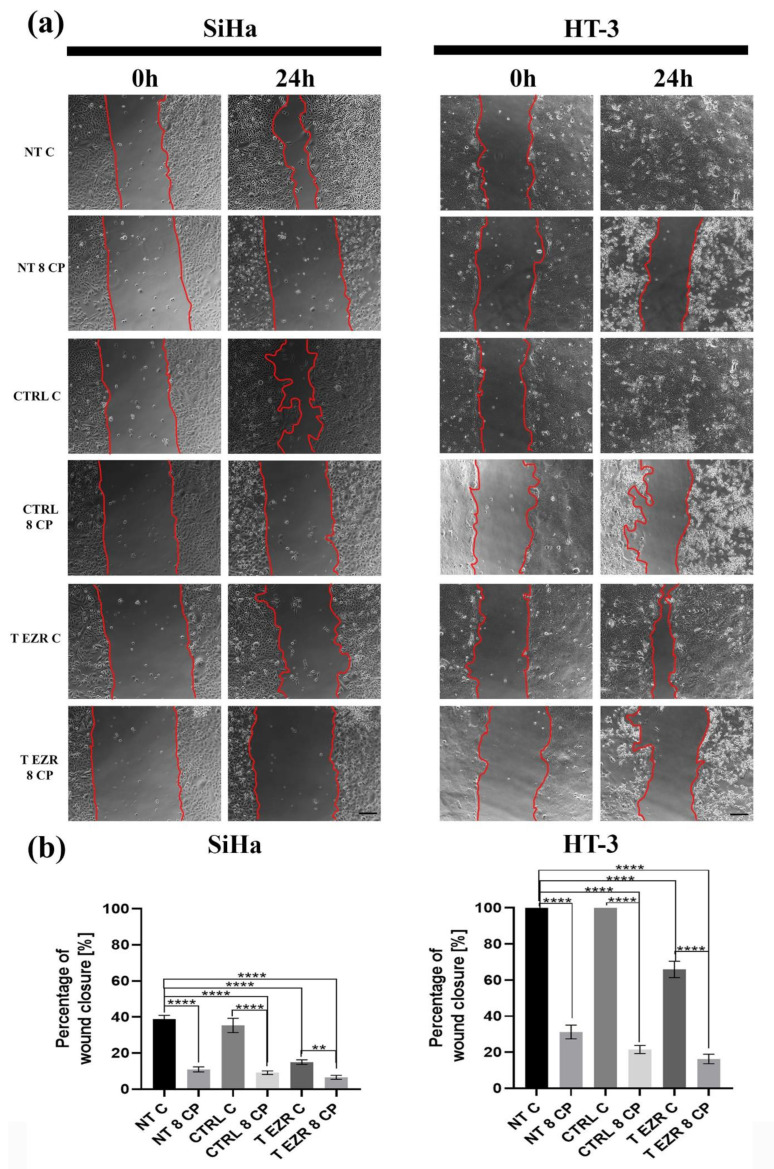
Wound healing assay. (**a**) Representative images of the wound healing assay for SiHa and HT-3 cells at the time of scratching (0 h) and after 24 h. (**b**) Statistical analysis of the average results for the percentage of healing of the wound surface after 24 h. A closed wound was considered 100%. NT—cells without transfection, CTRL—cells with control siRNA, T EZR—cells with siRNA directed against EZR, C—untreated cells, and 8 CP—cells treated with 8 µM CP. The average result of the tests. Statistically significant differences were marked as ‘**’ *p* < 0.01, and ‘****’ *p* < 0.0001.

The Transwell migration and invasion assay confirmed that cells with lower levels of EZR have greater difficulty crossing the barrier created by the insert/Matrigel, respectively (Figure 5a,b). In the migration test for SiHa, we observed a reduction of cells numbered from 286 (NT C) to 84 (T EZR C) and for HT-3 decreased from 222 to 75 cells. Similarly, the invasion assay showed changes between NT C and T EZR C in the range from 58 to 37 cells for SiHa and from 66 to 13 cells for HT-3 (Figure 5a,b). In the next experiment, we showed that protein reduction resulted in decreased adhesion properties of the SiHa and HT-3 cells, especially at the initial time points after cell seeding. After 30 min, we observed a difference in the number of cells attached to the well in the range of 191 cells for SiHa NT C and 110 cells for SiHa T EZR C and 93 for HT-3 NT C and 55 for HT-3 T EZR C. Moreover, after 6 h, two-fold and three-fold lower number of T EZR C cells adhered to the well surface compared to non-transfected NT C cells were observed for SiHa and HT-3, respectively (Figure 6a,b). In all observations, the reduction of the migration potential was enhanced by the use of 8 µM CP concentration. The wound healing assay showed that the rate of free space closure is statistically significantly slower in EZR T cells than in NT and CTRL cells for both lines (Figure 7a,b). After 24 h of observation, the wound area for SiHa cells was covered by 38.84% (NT C), 35.37% (CTRL C), and 15% (T EZR C) and, for HT-3, 100% for C NT and CTRL and 65.97% for T EZR C. Additionally, the application of 8 µM CP intensified the noticeable effect (Figure 7b).

### 2.4. Fluorescent Staining of Selected Proteins

**Figure 8:**
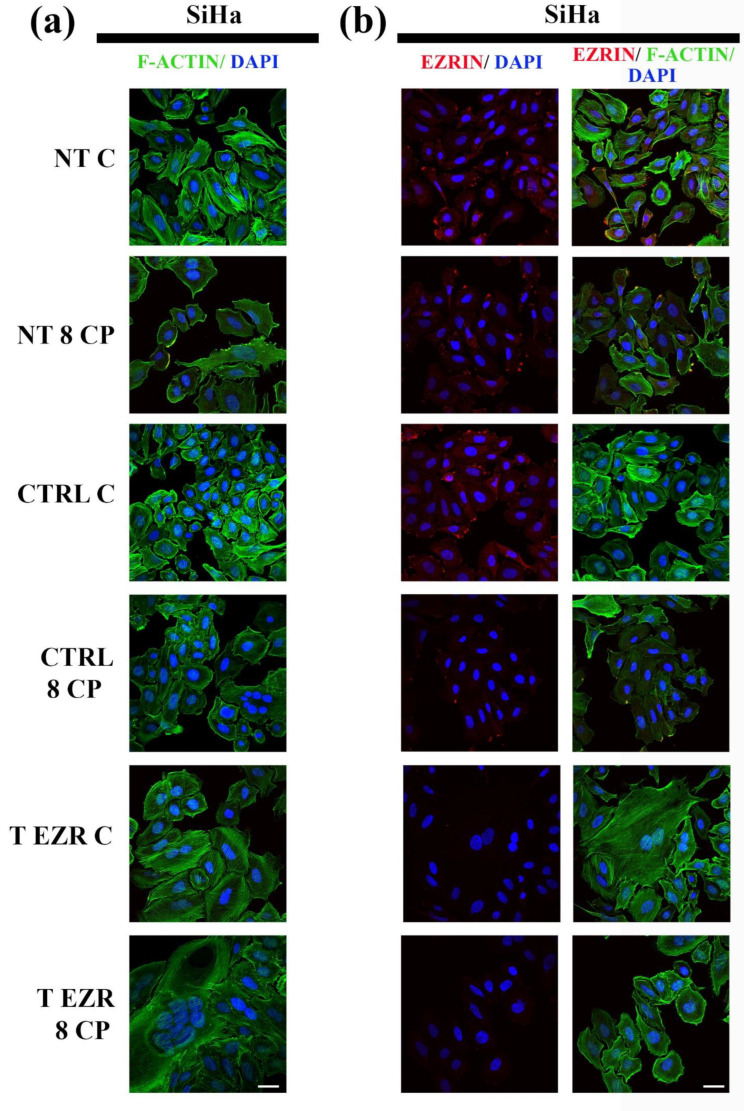
Fluorescent labeling of proteins in SiHa cells. (**a**) Representative images of F-actin (green) and cell nuclei (blue). (**b**) Representative images of EZR (red), F-actin (green), and cell nuclei (blue). NT—cells without transfection, CTRL—cells with control siRNA, T EZR—cells with siRNA directed against EZR, C—untreated cells, and 8 CP—cells treated with 8 µM CP. Bar = 50 µm.

**Figure 9:**
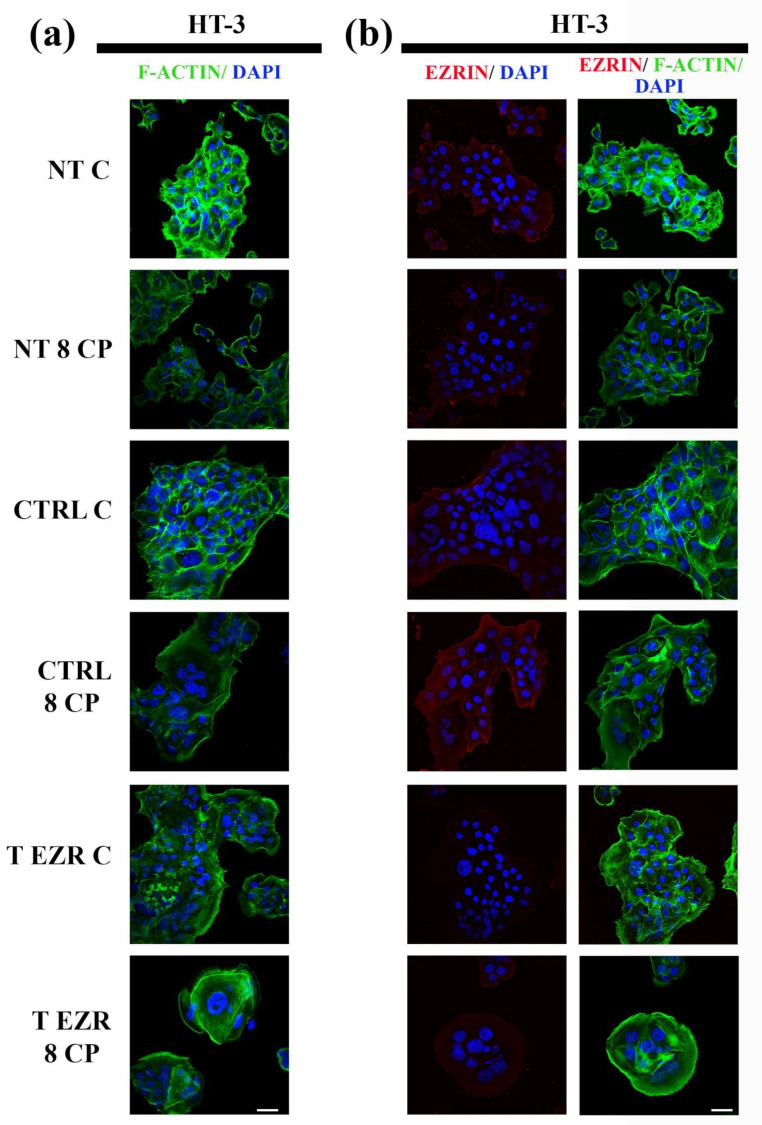
Fluorescent labeling of proteins in HT-3 cells. (**a**) Representative images of F-actin (green) and cell nuclei (blue). (**b**) Representative images of EZR (red), F-actin (green), and cell nuclei (blue). NT—cells without transfection, CTRL—cells with control siRNA, T EZR—cells with siRNA directed against EZR, C—untreated cells, and 8 CP—cells treated with 8 µM CP. Bar = 50 µm.

We decided to check whether reducing the level of EZR affects the organization of cytoskeletal proteins in SiHa and HT-3 cells. The limitation of motor and adhesive capabilities of SiHa and HT-3 cells resulting from the EZR level knockdown caused changes in the arrangement of F-actin (Figure 8a,b and Figure 9a,b). Analysis of the microscopic images of F-actin revealed two dominant patterns of proteins. In the case of SiHa cells, large cells with many micronuclei were visible, in which F-actin accumulated in the form of thick stress fibers in the cortical part of the cell. We assume that these were cells with a mitotic catastrophe phenotype (Figure 8a,b). The second observed cell type was characterized by shrunken cytoplasm and, as a result, a highly condensed protein (Figure 8a). In turn, of HT-3 cells, the downregulation of EZR resulted in the presence of smaller clusters of cells, in which F-actin accumulated in the form of a rim. Additionally, single cells were characterized by microfilaments in the form of short polymers located throughout the cytoplasm (Figure 9a,b). In addition, microscopic images showed a visible decrease in the fluorescence intensity of microfilaments as a result of CP treatment for both SiHa and HT-3 cells (Figure 8 and Figure 9).

Fluorescence microscopy was also used to assess EZR and confirmed the efficacy of the transfection. Major protein accumulation was seen at cell–cell contacts and motor processes (NT and CTRL cell populations of SiHa and HT-3). In EZR T cells of both cervical cancer cell lines, a significant decrease in protein signal was observed, with particular emphasis on cell–cell interactions (Figure 8b and Figure 9b).

## 3. Discussion

As is known, cell migration is closely related to a cascade of changes both in the cell itself and in the surrounding environment. An important role in this process is played by the cytoskeleton, with particular emphasis on actin filaments and its accompanying proteins. There are many classes of actin-binding proteins (ABP) that condition cell remodeling. In the context of motor capabilities, the ezrin/radixin/moesin (ERM) complex is important, playing a role in connecting microfilaments with the cell membrane [1]. The level of EZR depends on the physiological state of the cell. In normal tissues, it determines such processes as proliferation, morphogenesis, and migration. In the case of cancer cells, the level of the protein increases and, together with EMT markers (e.g., N-cadherin), promotes invasive features. Low levels of EZR correlate with increased expression of E-cadherin and a simultaneous decrease in β-catenin levels [10]. Given the cytoplasmic and membrane localization of EZR and the complexity of metastasis, its downregulation may have a dual effect on limiting metastasis. The membrane-localized protein is important for cell migration, through correlations with various proteins, e.g., integrins or the formation of motor structures. In turn, the cytoplasmic pool of EZR plays a role in stabilizing microfilaments [11]. The increased levels of EZR have been documented in various types of cancer [12,13]. Numerous reports also indicate the importance of protein in female cancers such as ovarian, breast, cervical, and endometrial cancer [13]. Li et al. (2021) conducted studies on ovarian cancer lines SKOV3 and CaOV3. Their results showed that increasing the level of EZR causes an increase in the level of vimentin and a decrease in E-cadherin in both tested lines [14]. In contrast, silencing the protein had the opposite effect [14]. Similar conclusions were described by Li et al. (2019) based on studies conducted on breast cancer lines MDA-MB-231 and MCF-7. Furthermore, they demonstrated high levels of EZR in breast cancer (BC) tissues and a correlation between EZR and the AKT pathway in BC [15]. In turn, Salama and Khairy (2023) and Ahmed et al. (2020) showed elevated levels of EZR in the immunohistochemical staining of endometrial cancer tissues [16,17].

In our work, we focused on assessing the effect of the downregulation of the EZR protein level in the context of reducing the metastatic potential of cervical cancer cells. It is known that cervical cancer, next to breast cancer, is one of the main problems in female oncology. Late detection of the disease and the formation of metastases significantly contribute to the deterioration of statistics among patients [18].

In the study, we used two types of cervical cancer cells differing in their genetic and morphological profiles. The first was isolated from a post-primary lesion (SiHa cell line) and then lymph node metastasis (HT-3). The aim of the study was to determine the response of these cells to reduced EZR levels in the context of migration potential and sensitivity to cytostatics. Our results, already at the stage of morphological observation and organization of the cytoskeleton showed significant differences between untransfected cells and those with reduced protein levels. SiHa cells were less sensitive to siRNA than HT-3. In addition, microscopic observations showed the appearance of cells with a mitotic catastrophe phenotype and a greater number of stress fibers. In the case of HT-3 with reduced EZR levels, a tendency to form fewer clusters was noted, as well as short F-actin fibers as the dominant pattern of microfilaments. We suggest that the mechanism of ezrin’s influence on the actin cytoskeleton may be multifaceted (i) by influencing the dynamics of F-actin polymerization and depolymerization and indirectly by modifying the cell shape and (ii) by regulating signaling pathways associated with the cell surface, as well as (iii) by interacting with other proteins responsible for contact with the cellular microenvironment. Our assumptions are consistent with the literature reports on the correlation of EZR with proteins such as integrins and cadherins [19]. Our panel of tests assessing cell motor properties showed that reducing the EZR levels significantly reduced the metastatic potential of SiHa and HT-3 cells. Similar results were obtained by Xi and Tang (2020), who also reduced the protein level in SiHa and CaSki cervical cancer cells [12]. Li et al. (2021), based on a panel of migration assays on ovarian cancer cells (SKOV3 and CaOV3), showed that EZR downregulation limited the migratory potential of ovarian cancer cells, while upregulation enhanced it [14]. In turn, Li et al. (2008) described that, after EZR silencing, breast cancer cells attained epithelial morphology and lost motor skills, while overexpression of the protein promoted a migratory phenotype [20]. Similar observations apply not only to female cancers but also to other types. The research by Tang et al. (2019) showed that downregulation of EZR in nasopharyngeal carcinoma significantly inhibited the migration of C6661 and NPC 6–10B cells [21].

Routine treatment for cervical cancer includes surgery (early stages), radiochemotherapy (locally advanced stages), and chemotherapy (metastatic or recurrent cancer) [22]. Among the cytotoxic drugs used in therapy, paclitaxel, CP, topotecan, gemcitabine, and vinorelbine are mentioned. The most frequently recommended treatment regimen in clinical practice is two-drug therapy and a combination of CP with one of the above drugs [23]. Due to its platinum content, CP has been an effective cytostatic agent in various types of cancer for many decades [24,25]. The spectrum of the drug’s action is based primarily on interaction with the DNA of cancer cells, inhibition of division, and as a further consequence, directing them to apoptosis. However, the literature also provides other aspects of CP’s cytotoxicity based on, for example, the induction of oxidative stress and various types of death (autophagy, nephroptosis, and mitotic catastrophe) or mitochondrial dysfunction [26]. One of the goals of our research was to assess the sensitivity of SiHa and HT-3 cells with reduced levels of EZR expression to cytostatics. Given the primary of CP in the treatment of cervical cancer, this drug was selected for our further studies. We describe, for the first time, the relationship between the reduction of EZR expression level in SiHa and HT-3 cells and cytostatic action. In the first step, we used the MTT assay, which allowed us to select the dose and assess the cytotoxicity of the drug on the tested cell lines. When choosing the final concentration (8 µM CP), we were guided by the results of the SiHa cell line. According to the literature, the cell population originating from the primary tumor is many times more resistant to drug treatment than metastases in various types of cancer [27]. This is due to the fact that the cells that form metastases are less heterogeneous than the primary tumors, and to a large extent, they are dominated by cells in the phase of intensive division, which is the target of cytostatics. Moreover, thanks to the blood supply, metastases are characterized by better drug availability [28,29]. Additionally, it is worth considering genetic aspects, such as differences in the expression level of genes related to drug transport or resistance mechanisms [30]. The results documented in our work indicate that HT-3 cells are more sensitive to CP than SiHa. The use of the drug on transfected cells resulted in an increase in the compound’s cytotoxicity and the percentage of apoptotic cells compared to non-transfected cells. We also observed a change in the proliferation rate to the detriment of the transfected cells. In both cases, SiHa and HT-3 cells after treatment with CP had lower invasive and clonogenic properties. Based on the literature, we suggest that the differences observed between the SiHa and HT-3 cell lines in basic vital processes after CP treatment may be a consequence of different responses of the signaling pathways of cells from primary and metastatic lesions. Examples include the Akt/mTOR, PI3K/Akt, and MAPK pathways, which are associated with cell survival and sensitivity to therapies [8,31,32]. The literature provides correlations between EZR levels and the above pathways, which may additionally explain the intensity of the observed cytotoxic effect [4,15]. Comparing all the above data between non-transfected cells and those with reduced EZR levels, we suggest that reducing the protein level in cervical cancer cells increases their sensitivity to CP. Similar results were reported by Chen et al. (2013), who observed that lung cancer cells were more sensitive to the action of CP, docetaxel, pirarubicin, and gemcitabine after EZR reduction [33].

## 4. Materials and Methods

### 4.1. Cell Culture and Treatment

Cervical cancer cell lines SiHa (HTB-35™) isolated from primary uterine tissue and HT-3 (HTB-32™) isolated from the lymph node of the female with cervical carcinoma were purchased from American Type Culture Collection (ATCC, Manassas, VA, USA). Cells were cultured under standard conditions (37 °C, 5% CO_2_, 95% humidity) with Eagle’s Minimum Essential Medium (EMEM, Corning, New York, NY, USA) and McCoy’s 5A Medium respectively, supplemented with 10% Fetal Bovine Serum (FBS, Corning, New York, NY, USA) and 1% Penicillin–Streptomycin Solution (P/S, Corning, New York, NY, USA). The cells were cultured to the sixth passage in T-25 cm^2^ flasks (Thermo Fisher Scientific, Waltham, MA, USA), or 24-, 12-, and 6-well plates (Merck KGaA, Darmstadt, Germany), depending on the experiment. Cells in the culture were monitored for mycoplasma, conducted by fluorescent staining using 4′,6-diamidino-2-phenylindole dihydrochloride-staining solution (DAPI, Merck KGaA, Darmstadt, Germany) [34].

To assess the effect of EZR downregulation on the sensitivity of cervical cancer cells, treatment with standard cytostatic CP was used. The concentration of the drug used in the study was selected experimentally on the basis of MTT tests described in our previous studies [35]. From the analyzed results of the use of cisplatin (CP) in doses of 2–10 µM, 8 µM was selected for further experiments.

### 4.2. Cell Transfection

EZR was downregulated in both cervical cancer cells using siRNA (Santa Cruz Biotechnology, Dallas, TX, USA) and the SE Cell Line 4D-Nucleofector^®^X kit (Lonza, Basel, Switzerland) by nucleofector electroporation. Transfection was performed using the CM-137 pulse program for SiHa and cells HT-3 cells (T EZR). The efficiency was evaluated by pmaxGFP plasmid included in the transfection kit, using the Tali^®^ Image-based cytometer (Invitrogen Life Technologies, Carlsbad, CA, USA) and by microscopic analysis of the cells using the Nikon Eclipse E800 fluorescence microscope and NIS-Elements 4.0 software (Nikon, Tokyo, Japan). The level of EZR downregulation was analyzed by Western Blot using anti-Ezrin antibody (mouse, monoclonal, 1:750, Sigma-Aldrich, Darmstadt, Germany), Anti-GADPH (1:1000; Santa Cruz Biotechnology, Dallas, TX, USA), and secondary anti-mouse antibody (1:4000, Santa Cruz Biotechnology, Dallas, TX, USA), following the procedure described in our previous articles [36]. Data for statistical evaluation were obtained based on densiometric measurements of bands obtained by Western Blot. Non-transfected cells (NT) and cells transfected with control siRNA (CTRL; Santa Cruz Biotechnology, Dallas, TX, USA) were used as controls. After 72 h (h), the cells were treated with CP for 24 h and used for further experiments.

### 4.3. Cell Death

The annexin V/propidium iodide (AV/PI) double-staining method described in detail in our earlier article was used to assess the type of cell death [29]. The studies assessed the type of cell death in SiHa and HT-3 non-transfected cells with control siRNA and downregulation of EZR treated/not treated with CP. An apoptosis assay kit that contains AV Alexa Fluor 647, propidium iodide (PI), and annexin-binding buffer (ABB) (Elabscience^®^, Houston, TX, USA) was used according to the manufacturer’s instructions.

### 4.4. Colony Formation Assay

To assess clonogenicity, SiHa and HT-3 NT, CTRL, and T EZR (with/without 8 µM CP) cells were cultured under standard conditions for 14 days at a density of 1000 cells/well (6-well plate). Then, cells were fixed (4% PFA, 10 min, RT), incubated with methanol (100%, 20 min, RT), and visualized (0.4% gentian violet, 15 min, RT, dark; HASCO-LEK S.A, Wrocław, Poland). The colonies were photographed with the ChemiDoc MP Imaging System (Bio-Rad) and counted in ImageJ 1.45s (NIH).

### 4.5. Fluorescence Staining of Proteins

Fluorescent labeling of the proteins was used to visualize EZR and F-actin. Untransfected SiHa and HT-3 cells, with control plasmid and reduced EZR levels, were cultured on slides in a 12-well plate. Both control and CP-treated cells were fixed with 4% PFA. Non-specific proteins were blocked with 1% BSA (1 h, RT, Sigma Aldrich St. Louis, MO, USA), in which solution, primary antibodies were prepared (anti-ezrin, mouse monoclonal, 1:100, 1 h, RT, Invitrogen Life Technologies, Carlsbad, CA, USA). After 1 h, the cells washed with PBS (3 × 5 min, RT) were incubated with the secondary antibody (Alexa Fluor 564, 1:200, 1 h, RT, in the dark, Life Technologies, Carlsbad, CA, USA. F-actin was visualized with Alexa Fluor 488 phalloidin (1:40, 20 min, RT, in the dark, Invitrogen, Life Technologies, Carlsbad, CA, USA), while cell nuclei were treated with DAPI (1:1000; 10 min, RT, in the dark; Sigma Aldrich, St Louis, MO, USA), in which solution, primary antibodies were prepared (anti-ezrin, mouse monoclonal). After a series of washes, the cells prepared in this way were closed with PolyAquaMount. The preparations were assessed in a Nikon Eclipse E800 fluorescence microscope and NIS-Elements 4.0 software (Nikon) and a C1 confocal microscope (Nikon, Tokyo, Japan) with a 60× oil immersion objective and Nikon EZ-C1 software (Nikon, Tokyo, Japan).

### 4.6. Transwell Migration and Invasion Assay

Cells of all groups were seeded in medium with 1% FBS at a density of 0.5 × 10^5^/upper site of 0.8 um insert (Corning, New York, NY, USA). The wells of the 24-well plates in which the inserts were placed contained medium with 20% FBS. The cells were incubated under standard conditions for 24 h. In the invasion test, the same procedure was used, enriched with the preparation of a Matrigel monolayer in the inner part of the insert. In both tests, after 24 h, the inserts were removed, and the cells were fixed with 4% PFA (10 min, RT) treated with 100% methanol (20 min, RT) and visualized with 0.4% gentian violet (15 min, RT, in the dark; HASCO-LEK S.A, Wrocław, Poland). Documentation was made using a light microscope Nikon Eclipse E800 (Nikon) and CCD camera DS-5Mc-U1 and NIS-Elements software version 3.30 (Nikon) and ImageJ (NIH). For statistical analysis, cells were counted and averaged.

### 4.7. Cell Adhesion Assay

SiHa and HT-3 cells (NT, CTRL, and T EZR) were seeded in a 12-well plate without and after CP treatment at a density of 0.2 × 10^5^ cells/well. The adhesion properties of the cells were assessed at time points 0.5, 1, 1.5, 2, and 6 h after seeding. Pictures of each well were taken in randomly selected places using an Olympus CKX53 inverted light microscope (Evident Microscopy Technologies, Shinjuku-ku, Tokyo, Japan). The number of cells attached to the well surface for each time was assessed in ImageJ (NIH) and compared to untreated cells (NT).

### 4.8. Wound-Healing Assay

Untransfected cells, EZR siRNA-transfected cells, and CTRL SiHa and HT-3 cells were cultured to form monolayers. Then, the surface of the well (24-well plate) was mechanically damaged with a tip, and some of the cells were treated with 8 µM CP. The wound healing test involved observing the healing of the wound under an inverted motorized microscope (Zeiss) equipped with a live cell imaging incubation system (Pecon). The imaging setup used an EC Plan-Neofluar 10×/0.30 Ph1 air objective lens, an Axiocam 503 monaural camera, and ZEN 2 software (all from Zeiss). Pictures were taken from selected points for each well at 10-min intervals for 24 h. The obtained images were analyzed for parameters characteristic of the wound healing assay in ImageJ (NIH) and subjected to statistical analysis.

### 4.9. Statistical Analysis

The selection of statistical tests (parametric and non-parametric) was based on the results of the Shapiro–Wilk test assessing the distribution of samples (normal and non-normal distribution). In the case of normal distribution, charts showing the mean with +/− SD were used, while, for the others, the median was presented. For MTT analysis, the Wilcoxon signed-rank test was used. To evaluate the cell death, colony formation, and migration/invasion, ordinary one-way analysis of variance with Dunnett’s or Tukey’s multiple comparison test was used. For wound healing assay analysis, two-way analysis of variance with Sidak’s multiple comparison test was performed. All statistical analysis was performed in GraphPad Prism 6 Software. Significance was presented in the figures as * *p* < 0.05, ** *p* < 0.01, *** *p* < 0.001, or **** *p* < 0.0001.

## 5. Conclusions and Future Perspective

In conclusion, we confirm that the reduction in EZR expression level in both primary lesion and lymph node metastasis cells significantly reduces the metastatic potential of cervical cancer cells. Moreover, we found for the first time that the reduction of the protein level sensitizes cells to cytostatic action such as CP and thus also enhances the antimigratory effect. The above conclusions confirm that EZR may be an effective aspect in limiting metastasis. In this context, other proteins from the ERM complex, as well as integrins and cadherins, are also of interest, which can potentially interact synergistically with EZR. Moreover, in the long term, an interesting aspect of research may be to understand the mechanisms determining changes in F-actin and the sensitivity of cervical cancer cells to CP under conditions of reduced EZR expression.

## Data Availability

The original contributions presented in the study are included in the article, further inquiries can be directed to the corresponding author.

## References

[1] Nieto M.A., Huang R.Y.-J., Jackson R.A., Thiery J.P. (2016). EMT: 2016. Cell.

[2] Ganesh K., Massagué J. (2021). Targeting Metastatic Cancer. Nat. Med..

[3] Barik G.K., Sahay O., Paul D., Santra M.K. (2022). Ezrin Gone Rogue in Cancer Progression and Metastasis: An Enticing Therapeutic Target. Biochim. Biophys. Acta BBA Rev. Cancer.

[4] Buenaventura R.G.M., Merlino G., Yu Y. (2023). Ez-Metastasizing: The Crucial Roles of Ezrin in Metastasis. Cells.

[5] Fröse J., Chen M.B., Hebron K.E., Reinhardt F., Hajal C., Zijlstra A., Kamm R.D., Weinberg R.A. (2018). Epithelial-Mesenchymal Transition Induces Podocalyxin to Promote Extravasation via Ezrin Signaling. Cell Rep..

[6] Song Y., Ma X., Zhang M., Wang M., Wang G., Ye Y., Xia W. (2020). Ezrin Mediates Invasion and Metastasis in Tumorigenesis: A Review. Front. Cell Dev. Biol..

[7] Yu Z., Sun M., Jin F., Xiao Q., He M., Wu H., Ren J., Zhao L., Zhao H., Yao W. (2015). Combined Expression of Ezrin and E-Cadherin Is Associated with Lymph Node Metastasis and Poor Prognosis in Breast Cancer. Oncol. Rep..

[8] Quan C., Sun J., Lin Z., Jin T., Dong B., Meng Z., Piao J. (2019). Ezrin Promotes Pancreatic Cancer Cell Proliferation and Invasion through Activating the Akt/mTOR Pathway and Inducing YAP Translocation. Cancer Manag. Res..

[9] Cancer of the Cervix Uteri—Cancer Stat Facts. https://seer.cancer.gov/statfacts/html/cervix.html.

[10] Chen M.-J., Gao X.-J., Xu L.-N., Liu T.-F., Liu X.-H., Liu L.-X. (2014). Ezrin Is Required for Epithelial-Mesenchymal Transition Induced by TGF-Β1 in A549 Cells. Int. J. Oncol..

[11] Li L.-Y., Xie Y.-H., Xie Y.-M., Liao L.-D., Xu X.-E., Zhang Q., Zeng F.-M., Tao L.-H., Xie W.-M., Xie J.-J. (2017). Ezrin Ser66 Phosphorylation Regulates Invasion and Metastasis of Esophageal Squamous Cell Carcinoma Cells by Mediating Filopodia Formation. Int. J. Biochem. Cell Biol..

[12] Xi M., Tang W. (2020). Knockdown of Ezrin Inhibited Migration and Invasion of Cervical Cancer Cells in Vitro. Int. J. Immunopathol. Pharmacol..

[13] Lipreri da Silva J.C., Vicari H.P., Machado-Neto J.A. (2023). Perspectives for Targeting Ezrin in Cancer Development and Progression. Future Pharmacol..

[14] Li M.J., Xiong D., Huang H., Wen Z.Y. (2021). Ezrin Promotes the Proliferation, Migration, and Invasion of Ovarian Cancer Cells. Biomed. Environ. Sci..

[15] Li N., Kong J., Lin Z., Yang Y., Jin T., Xu M., Sun J., Chen L. (2019). Ezrin Promotes Breast Cancer Progression by Modulating AKT Signals. Br. J. Cancer.

[16] Salama M.E.M., Khairy D.A. (2023). Moesin and Ezrin as New Promising Markers for Early Detection of Endometrial Carcinoma: An Immunohistochemical Study. Asian Pac. J. Cancer Prev..

[17] Ahmed A.R.H., Yousef G.M., Abdel-Raheem M.S.E., Muhammad E.M.S. (2020). A Potential Invasion-Promoting Role for Ezrin in Endometrial Carcinoma. Res. Oncol..

[18] Kong J., Li Y., Liu S., Jin H., Shang Y., Quan C., Li Y., Lin Z. (2013). High Expression of Ezrin Predicts Poor Prognosis in Uterine Cervical Cancer. BMC Cancer.

[19] Zacapala-Gómez A.E., Navarro-Tito N., Alarcón-Romero L.D.C., Ortuño-Pineda C., Illades-Aguiar B., Castañeda-Saucedo E., Ortiz-Ortiz J., Garibay-Cerdenares O.L., Jiménez-López M.A., Mendoza-Catalán M.A. (2018). Ezrin and E-Cadherin Expression Profile in Cervical Cytology: A Prognostic Marker for Tumor Progression in Cervical Cancer. BMC Cancer.

[20] Li Q., Wu M., Wang H., Xu G., Zhu T., Zhang Y., Liu P., Song A., Gang C., Han Z. (2008). Ezrin Silencing by Small Hairpin RNA Reverses Metastatic Behaviors of Human Breast Cancer Cells. Cancer Lett..

[21] Tang Y., Sun X., Yu S., Bie X., Wang J., Ren L. (2019). Inhibition of Ezrin Suppresses Cell Migration and Invasion in Human Nasopharyngeal Carcinoma. Oncol. Lett..

[22] Burmeister C.A., Khan S.F., Schäfer G., Mbatani N., Adams T., Moodley J., Prince S. (2022). Cervical Cancer Therapies: Current Challenges and Future Perspectives. Tumour Virus Res..

[23] Marth C., Landoni F., Mahner S., McCormack M., Gonzalez-Martin A., Colombo N. (2017). Cervical Cancer: ESMO Clinical Practice Guidelines for Diagnosis, Treatment and Follow-Up. Ann. Oncol..

[24] Dasari S., Tchounwou P.B. (2014). Cisplatin in Cancer Therapy: Molecular Mechanisms of Action. Eur. J. Pharmacol..

[25] Nguyen V.T., Winterman S., Playe M., Benbara A., Zelek L., Pamoukdjian F., Bousquet G. (2022). Dose-Intense Cisplatin-Based Neoadjuvant Chemotherapy Increases Survival in Advanced Cervical Cancer: An Up-to-Date Meta-Analysis. Cancers.

[26] Elmorsy E.A., Saber S., Hamad R.S., Abdel-Reheim M.A., El-Kott A.F., AlShehri M.A., Morsy K., Salama S.A., Youssef M.E. (2024). Advances in Understanding Cisplatin-Induced Toxicity: Molecular Mechanisms and Protective Strategies. Eur. J. Pharm. Sci..

[27] Maniwa Y., Yoshimura M., Hashimoto S., Takata M., Nishio W. (2010). Chemosensitivity of Lung Cancer: Differences between the Primary Lesion and Lymph Node Metastasis. Oncol. Lett..

[28] Fidler I.J. (2003). The Pathogenesis of Cancer Metastasis: The “seed and Soil” Hypothesis Revisited. Nat. Rev. Cancer.

[29] Joyce J.A., Pollard J.W. (2009). Microenvironmental Regulation of Metastasis. Nat. Rev. Cancer.

[30] Zhu H., Luo H., Zhang W., Shen Z., Hu X., Zhu X. (2016). Molecular Mechanisms of Cisplatin Resistance in Cervical Cancer. Drug Des. Devel Ther..

[31] Stefani C., Miricescu D., Stanescu-Spinu I.-I., Nica R.I., Greabu M., Totan A.R., Jinga M. (2021). Growth Factors, PI3K/AKT/mTOR and MAPK Signaling Pathways in Colorectal Cancer Pathogenesis: Where Are We Now?. Int. J. Mol. Sci..

[32] Zhang L., Wu J., Ling M.T., Zhao L., Zhao K.-N. (2015). The Role of the PI3K/Akt/mTOR Signalling Pathway in Human Cancers Induced by Infection with Human Papillomaviruses. Mol. Cancer.

[33] Chen Q.-Y., Xu W., Jiao D.-M., Wu L.-J., Song J., Yan J., Shi J.-G. (2013). Silence of Ezrin Modifies Migration and Actin Cytoskeleton Rearrangements and Enhances Chemosensitivity of Lung Cancer Cells in Vitro. Mol. Cell Biochem..

[34] Jung H., Wang S.-Y., Yang I.-W., Hsueh D.-W., Yang W.-J., Wang T.-H., Wang H.-S. (2003). Detection and Treatment of Mycoplasma Contamination in Cultured Cells. Chang. Gung Med. J..

[35] Hałas-Wiśniewska M., Zielińska W., Izdebska M., Grzanka A. (2020). The Synergistic Effect of Piperlongumine and Sanguinarine on the Non-Small Lung Cancer. Molecules.

[36] Izdebska M., Zielińska W., Krajewski A., Hałas-Wiśniewska M., Mikołajczyk K., Gagat M., Grzanka A. (2021). Downregulation of MMP-9 Enhances the Anti-Migratory Effect of Cyclophosphamide in MDA-MB-231 and MCF-7 Breast Cancer Cell Lines. Int. J. Mol. Sci..

